# Mental wellbeing of frontline health workers post-pandemic: lessons learned and a way forward

**DOI:** 10.3389/fpubh.2023.1204662

**Published:** 2023-06-19

**Authors:** Thorsten Grünheid, Ahmad Hazem

**Affiliations:** ^1^Division of Orthodontics, School of Dentistry, University of Minnesota, Minneapolis, MN, United States; ^2^Department of Hospitalist Medicine, Essentia Health, Duluth, MN, United States

**Keywords:** burnout, COVID-19, depression, mental health, stress, wellbeing

## Abstract

**Objective:**

To assess the state of mental wellbeing among medical and dental frontline health workers as the COVID-19 pandemic transitions to an endemic phase and to determine what employer-provided intervention strategies these workers perceive as effective and desirable to improve their mental wellbeing.

**Methods:**

An anonymous online survey distributed to frontline health workers in a hospitalist program of a tertiary care medical center and a university dental school in Minnesota in September 2022. The survey contained validated tools to measure depression severity, levels of perceived stress, and mental health status as well as questions to determine effective strategies to improve emotional wellbeing among these health workers. Data was evaluated on an aggregate level as well as stratified by level (e.g., physician, staff) and field (e.g., medicine, dentistry).

**Results:**

On average, all groups of health workers suffered from moderate to moderately severe depression, had a much higher perceived stress level than average, and had a fair mental health status. There were no significant differences in depression severity, stress level, or mental health status among physicians, dentists, medical staff, and dental staff. The majority of the respondents perceived adjusted work hours, rewards and incentives, and teamwork as the most effective and desirable strategies to improve their mental wellbeing.

**Conclusion:**

The current mental wellbeing of frontline health workers is poor. Many are dissatisfied with healthcare and consider leaving the industry. To improve their employees’ mental wellbeing, healthcare employers might want to consider adjusted work hours, rewards, and teamwork as these intervention strategies are perceived as most effective and desirable by the intended recipients.

## 1. Introduction

Worldwide, the physical and mental health of healthcare workers has been put at risk by COVID-19. The combined effects of severe morbidity and mortality, staff shortages and increased workloads, continuously changing policies and procedures, high exposure risk, and negative personal life events represent a virtual barrage of psychosocial stressors for these individuals (1). An impressive number of scientific publications have outlined how emerging problems added to the usual stressors of the medical profession, resulting in disorders such as post-traumatic stress, sleep problems, anxiety, depression, and burnout (2–4). For example, after the initial pandemic surge in Spring 2020, 14% of healthcare workers surveyed in a tertiary care center in Baltimore reported depression, with 43% reporting anxiety, 32% sleep disturbance, 22% post-traumatic stress disorder (PTSD), 22% depersonalization, 46% emotional exhaustion, and 23% lower resilience (5).

These findings are not unique to the United States. For example, in a large cross-sectional study evaluating pandemic impact on staff in three United Kingdom hospitals, nearly 60% had evidence of mental health disorders, more than 30% had evidence of PTSD, 27% of depression, 23% anxiety, and over 10% of alcohol abuse. Nearly 28% of those surveyed indicated that they had thoughts of taking their own life (6). Similar findings were reported from other countries including Germany, Ireland, China, South Korea, and Saudi Arabia (7).

Although COVID-19 no longer dominates our lives, the pandemic’s side effects, such as inflation, staffing shortages, and supply chain problems, are still presenting challenges for the healthcare industry (8, 9). Frontline workers are still processing the trauma of what they witnessed in the early days. This has led to substantial burnout. In Medscape’s 2021 National Physician Burnout and Suicide Report, 42% of physicians reported burnout and 47% reported a severe impact of their jobs on their personal lives (10). Nurses are similarly affected (11). This clinician burnout inevitably affects the quality of care and patient safety as it contributes to medical errors across different specialties. Clinicians with signs of burnout have been found to be twice as likely to make medical errors (12).

Perhaps even more disturbing is that burnout increases suicide risk. Each year, 400 physicians in the United States take their own lives (13). Suicide is the only cause of mortality that is higher in physicians than in non-physicians (14). Female physicians are at an especially high risk, and physicians in the United States are overall at a higher risk than their colleagues in the rest of the world (15).

An often-overlooked group of frontline healthcare workers affected by COVID-19 is dental personnel. Working up close on unmasked patients, and performing aerosol-generating procedures, they have an exceptionally high exposure risk. In the early days of the pandemic, their livelihoods were put at jeopardy as legislators imposed mandated clinic closures. It has been suggested that dentists’ levels of depression, anxiety, and stress have increased substantially during the COVID-19 pandemic, resulting in psychological distress, mental health issues, and burnout (16–18). However, objective data regarding their mental wellbeing is scarce.

Traditionally, burnout and loss of wellbeing were equated to a loss of resilience against stress. As a consequence, many employers have focused their efforts on improving resilience (19). However, a growing body of evidence suggests that there are no significant benefits of resilience interventions on depression and behavioral symptoms (20). In the pursuit of creating healthier workplaces, employers, including health systems, have shifted their efforts toward providing stress management interventions, encouraging social support, and implementing high-quality performance management (21). However, it remains unclear if these efforts to address stress and burnout are effective or desired by the recipients.

The aims of this study were to assess and compare the current state of mental wellbeing among medical and dental frontline health workers, and to determine what measures employers can take to effectively help and improve mental wellbeing. Specifically, we aimed at quantifying depression severity, perceived stress level, and mental health status among individuals with patient-facing duties two years into the COVID-19 pandemic. We also aimed at assessing what employer-provided intervention strategies these individuals perceive as effective and desirable to improve their mental wellbeing. The null hypothesis was that there are no significant differences in depression severity, perceived stress level, and mental health status between medical and dental frontline health workers.

## 2. Methods

This cross-sectional survey study was approved by the Institutional Review Boards at Essentia Health Duluth and the University of Minnesota (Study number 00016700). Potential subjects were all employees at Essentia Health’s Section of Hospital Medicine and the University of Minnesota School of Dentistry. Inclusion criteria were age above 18 years and appointment with patient-facing duties. Exclusion criteria were student or retired employee, or 100% research or administrative appointment. A total of 430 individuals were targeted as potential subjects, 82 at Essentia Health and 348 at the University of Minnesota School of Dentistry.

The subjects were contacted by email with a link to a custom-designed survey hosted by an online survey platform (Qualtrics, Provo, UT) in September 2022. The subjects were able to complete the survey anonymously on their computer or mobile device using the browser of their choice. No time restriction was imposed, and the subjects were able to save their responses and return to the survey if they chose not to complete it in one session. The survey was open for two consecutive weeks. Two reminder emails were sent to those who did not opt out from future communication.

**Figure 1:**
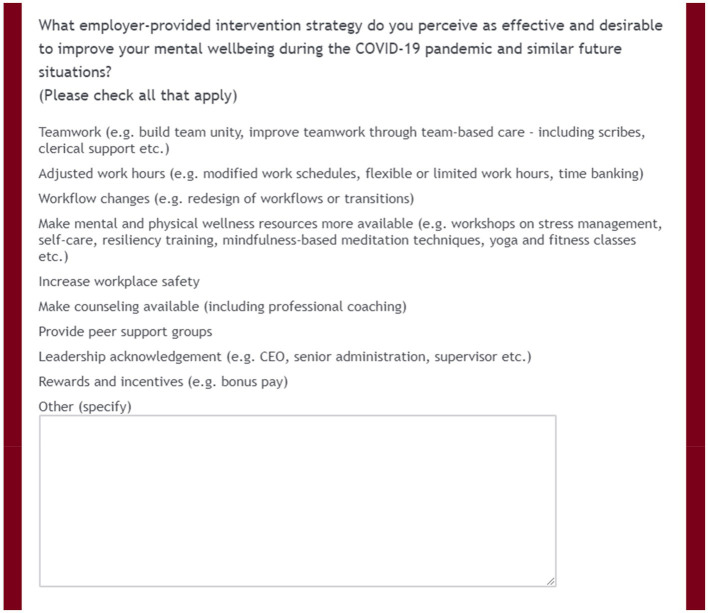
Description of employer-provided intervention strategies in the survey tool.

Following a brief introduction to the survey, a consent information form with phone numbers and hyperlinks to support organizations was presented as a downloadable PDF file. By proceeding to the survey, the subjects confirmed that they provided consent and wished to participate in the survey. The survey consisted of multiple-choice questions on subject demographics, number of years worked in healthcare, current role, time worked in direct patient contact since COVID-19 was declared a pandemic, the Patient Health Questionnaire 9 (PHQ-9), the Perceived Stress Scale 10 (PSS-10), the Patient-Reported Outcomes Measures Information System (PROMIS) Global-10, satisfaction with the current state of the healthcare industry and their career choice, as well as a question on employer-provided intervention strategies that the subjects perceive as effective and desirable to improve their mental wellbeing during the COVID-19 pandemic and similar future situations (Figure 1). The list of proposed interventions was compiled from existing studies. The evidence for these interventions has been summarized in several systematic reviews, which suggest moderate-to-weak evidence that primary, secondary, and combined interventions can reduce burnout and stress in healthcare workers (22–24). In addition, the subjects had the opportunity to provide free-text input.

The PHQ-9 is a validated tool to detect depression and gauge its severity (25). The PSS-10 is a validated tool to screen for stress and adds a temporal correlation to the respondents’ symptoms (26). The PROMIS Global-10 is a publicly available global health assessment tool to assess general domains of health and functioning including overall physical health, mental health, social health, pain, fatigue, and overall perceived quality of life (27). It allows derivation of a global physical health score and a global mental health score. These scores can be used to arrive at a summary of health and mental status (28), and can be converted to a T-score metric, which allows comparisons to a general population (29).

Once the survey was closed, the resultant data was downloaded and PROMIS Global-10 summary scores, global physical health summed raw scores, and global mental health summed raw scores were calculated. The global mental health summed raw scores were then converted into T-score values for each individual respondent. T-score distributions are standardized such that a score of 50 represents the mean for the United States general population, and the standard deviation around that mean is 10 points (26).

### 2.1. Statistical analysis

Summary statistics (mean, standard deviation) were calculated for all continuous variables (PHQ-9 scores, PSS-10 scores, and PROMIS global mental health T-scores). Categorical variables were summarized by counts and percentages. The survey data was stratified by role (e.g., physician, staff) and field (e.g., medicine, dentistry), and analyzed both on an aggregate level and on a group level. The normality of the data was assessed using Q-Q plots to visualize the normality and Shapiro–Wilk test when the Q-Q plots were not clear. Continuous variables were compared using Wilcoxon Rank Sum Test and Kruskal Wallis test since the normality assumption was not satisfied for some cases. Categorical variables were compared using Chi-square test or Fisher’s exact test when the sample size was less than five. Data obtained from attendees of the 2022 Minnesota State Fair between August 25 and September 5, 2022, using the same PROMIS Global-10 survey tool, served as a local non-healthcare control. All statistical analyses were performed using R Statistical Software 4.2.1 (R Foundation for Statistical Computing, Vienna, Austria) with *p*-values of less than 0.05 considered statistically significant.

## 3. Results

The overall survey response rate was 32% with response rates of 71% in the medical group and 23% in the dental group. The survey completion rate was 94%. Incomplete responses were excluded from further evaluation. Results are based on 130 complete responses.

Respondent demographics are shown in Table 1. The distributions of age and time in healthcare differed significantly between the medical and dental fields (both *p* < 0.001), most likely because of a number of residents in the dental group. There were no significant differences in gender distribution between the fields (*p* = 0.285). There were no significant differences in age or time in healthcare between males and females in either medical or dental fields (all *p* > 0.05).

**Table 1:** Respondent demographics.

Variable	Category	Medical	Dental	Overall
n		58	72	130
Age	18–34 yrs.	9 (15.5%)	22 (30.6%)	31 (23.8%)
	35–50 yrs.	39 (67.2%)	18 (25.0%)	57 (43.8%)
	51–65 yrs.	9 (15.5%)	20 (27.8%)	29 (22.3%)
	65+ yrs.	1 (1.7%)	12 (16.7%)	13 (10.0%)
Gender	Female	35 (60.3%)	51 (70.8%)	86 (66.2%)
	Male	23 (39.7%)	21 (29.2%)	44 (33.8%)
Time in healthcare	0–5 yrs.	6 (10.3%)	19 (26.4%)	25 (19.2%)
	6–10 yrs.	17 (29.3%)	12 (16.7%)	29 (22.3%)
	11–20 yrs.	21 (36.2%)	6 (8.3%)	27 (20.8%)
	21–30 yrs.	11 (19.0%)	16 (22.2%)	27 (20.8%)
	30+ yrs.	3 (5.2%)	19 (26.4%)	22 (16.9%)

Results are expressed as counts and percentages.

The respondents’ healthcare roles are shown in Table 2. Because of the small sample sizes of some groups, advanced practice providers, nursing staff, and other medical staff were pooled as ‘Medical staff’ for further analysis. Similarly, dental therapists/hygienists, dental assistants, and other dental staff were pooled as ‘Dental staff’ for further analysis. The majority of respondents (medical 96.6%; dental 95.8%; overall 96.2%) had worked for more than 6 months in direct patient contact during the COVID-19 pandemic. There were no significant differences between medical and dental fields (*p* = 1).

**Table 2:** Respondent healthcare roles.

Medical		Dental	
Physician	38 (29.2%)	Dentist	49 (37.7%)
Advanced practice provider	15 (11.5%)	Dental therapist/hygienist	8 (6.2%)
Nursing staff	1 (0.8%)	Dental assistant	4 (3.1%)
Other medical staff	4 (3.1%)	Other dental staff	11 (8.5%)

Results are expressed as counts and percentages.

The mean PHQ-9 scores of the various groups, indicating their depression severity, were as follows: Physicians 12.9 ± 4.7, Medical staff 15.1 ± 3.9, Dentists 14.3 ± 4.5, and Dental staff 14.3 ± 4.7. There were no significant differences among groups (*p* = 0.090). The mean scores suggest that, on average, all groups suffered from moderate to moderately severe depression. The distributions of depression severity within the groups together with proposed treatment actions (25) are shown in Table 3. Individuals with severe depression were predominantly female (72.2%), between ages 18 and 50 years (72.2%), and had worked in healthcare for 6–20 years (60.0%).

**Table 3:** Distribution of depression severity of provider groups together with proposed treatment actions (25).

PHQ-9 score	Depression severity	Physician	Medical staff	Dentist	Dental staff	Proposed treatment actions
0–4	None–minimal	0%	0%	0%	0%	None
5–9	Mild	23.7%	5%	18%	16%	Watchful waiting, repeat PHQ-9 at follow-up
10–14	Moderate	50%	45%	44%	40%	Treatment plan, considering counseling, follow-up and/or pharmacotherapy
15–19	Moderately severe	15.8%	35%	24%	28%	Active treatment with pharmacotherapy and/or psychotherapy
20–27	Severe	10.5%	15%	14%	16%	Immediate initiation of pharmacotherapy and, if severe impairment or poor response to therapy, expedited referral to a mental health specialist for psychotherapy and/or collaborative management

The mean PSS-10 scores of the various groups, indicating their perceived stress level, were as follows: Physicians 26.9 ± 3.9, Medical staff 31.3 ± 3.8, Dentists 29.7 ± 3.0, and Dental staff 28.8 ± 4.1. There were no significant differences among groups (*p* = 0.345). These mean scores suggest that, on average, all groups had a much higher perceived stress level than average. The distributions of perceived stress level within the groups together with associated health concern levels (26) are shown in Table 4. The vast majority of respondents (medical 100%; dental 98.6%; overall 99.2%) had much higher than average stress levels. There were no significant differences between medical and dental fields (*p* = 1).

**Table 4:** Distribution of perceived stress levels of provider groups together with associated health concern levels (26).

PSS-10 score	Perceived stress level	Physician	Medical staff	Dentist	Dental staff	Health concern level
0–7	Much lower than average	0%	0%	0%	0%	Very low
8–11	Slightly lower than average	0%	0%	0%	0%	Low
12–15	Average	0%	0%	0%	0%	Average
16–20	Slightly higher than average	0%	0%	0%	4%	High
21 and over	Much higher than average	100%	100%	100%	96%	Very high

The mean PROMIS Global-10 mental health T-scores of the various groups, indicating their mental health status, were as follows: Physicians 36.4 ± 10.4, Medical staff 43.0 ± 8.4, Dentists 40.3 ± 9.8, and Dental staff 39.8 ± 8.0. There were no significant differences among groups (*p* = 0.076). These mean scores suggest that, on average, the mental health status of all groups was fair. For comparison, the average score for the United States population is 50, suggesting good mental health (29). The distributions of mental health statuses within the groups are shown in Table 5. Health workers with poor mental health were predominantly between ages 35 and 50 years (41.3%) and had worked in healthcare for either less than 10 (37.0%) or more than 20 years (43.5%). Poor mental health affected both genders equally (male 45.7%, female 54.3%). A control group of 631 attendees of the 2022 Minnesota State Fair (196 male, 423 female, 12 prefer not to disclose; age 43.7 ± 17.7 years; range 18–102) had a PROMIS Global-10 mental health T-score of 53.2 ± 8.7 indicating good mental health. The distribution of mental health statuses within this group is shown in Table 5. A comparison with the groups of health workers showed statistically significantly lower mental health status of all groups of health workers than the non-healthcare control (*p* < 0.001).

**Table 5:** Distribution of mental health statuses of provider groups and a group of 2022 Minnesota State Fair attendees, which served as a local non-healthcare control.

T-score	Mental health	Physician	Medical staff	Dentist	Dental staff	State Fair control
65 and over	Excellent	0%	0%	0%	0%	12.8%
55–64.9	Very good	10.5%	0%	8.2%	0%	31.5%
45–54.9	Good	13.2%	40%	30.6%	26.1%	39.5%
35–44.9	Fair	23.7%	45%	24.5%	43.5%	14.6%
34.9 and below	Poor	52.6%	15%	36.7%	30.4%	1.6%

More than half of the respondents were satisfied with their career choice (yes 55%; no 14%; not sure 31%); however, only few were satisfied with the current state of the healthcare industry (yes 9%; no 65%; not sure 26%). One-third of the respondents would rather retire or do something else for a living if they had the opportunity (yes 33%; no 32%; not sure 35%).

**Figure 2:**
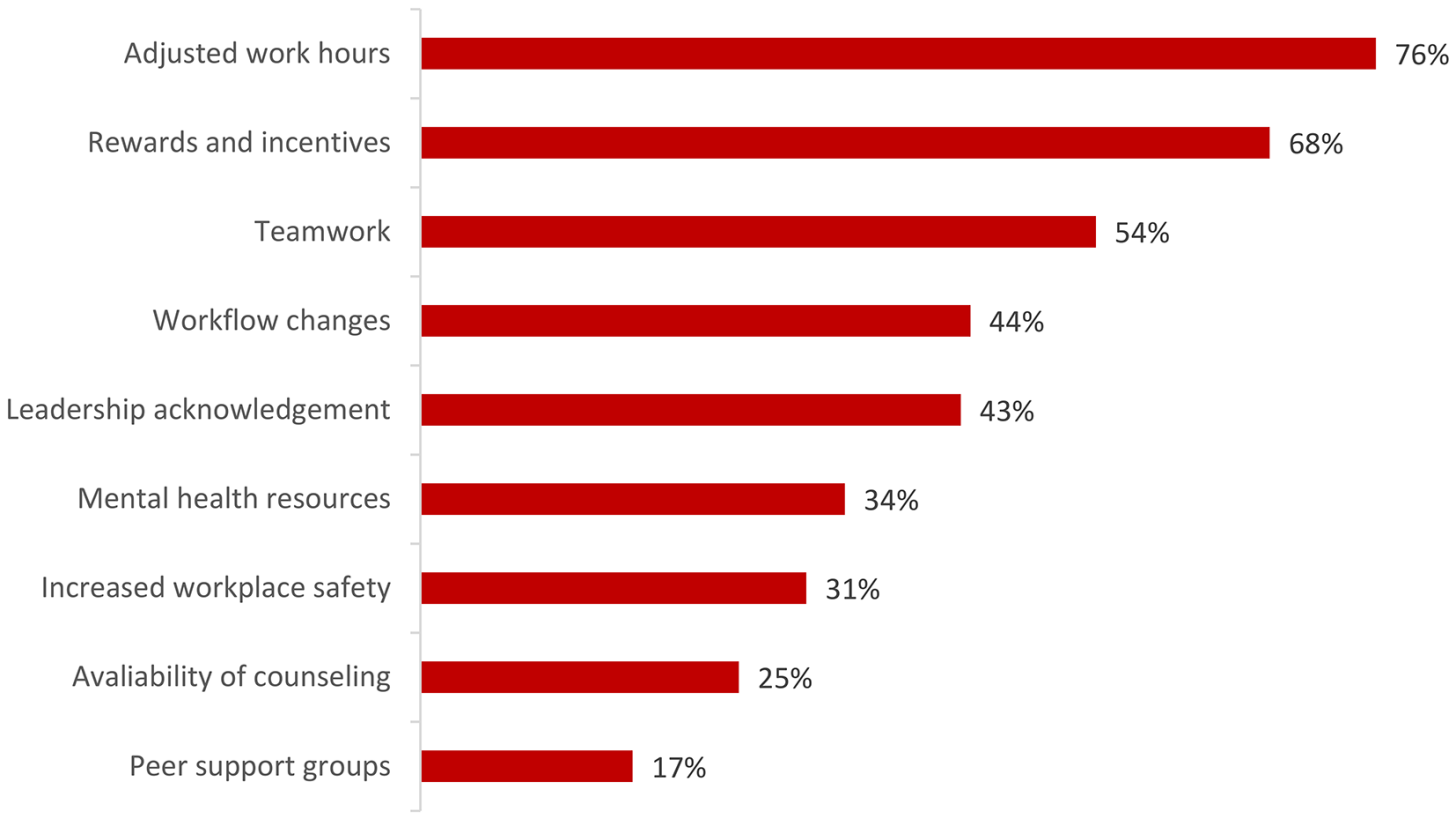
Employer-provided intervention strategies that the respondents perceived as effective and desirable to improve their mental wellbeing. More than one answer was possible per respondent.

The employer-provided intervention strategies that the subjects perceived as effective and desirable to improve their mental wellbeing are shown in Figure 2. More than half of the respondents perceived adjusted work hours (e.g., modified work schedules, flexible or limited work hours, time banking), rewards and incentives (e.g., bonus pay), and teamwork (e.g., build team unity, improve teamwork through team-based care; including scribes, clerical support etc.) as the most effective and desirable strategies. The interventions perceived as least effective and desirable were increased workplace safety, availability of counseling, and peer support groups.

## 4. Discussion

In 2020, 1,200,000 Americans attempted to take their own life, with nearly 46,000 of them seeing it through (30). Of those lost each year, approximately 400 are physicians with female physicians having a suicide death rate 250–400% higher than females in other professions. This appears to correlate with depression severity. Female physicians have a higher rate of major depression than age-matched females with doctorate level degrees (31). This female-dominant gender distribution is also reflected in the findings of the present study where respondents with severe depression were predominantly females between ages 18 and 50 years.

Unfortunately, the existing culture of medicine tends to give physician mental health and wellbeing low priority despite all the evidence that demonstrates its perils. In addition, healthcare workers, especially physicians, often defer self-care, resulting from concerns for their licensure, hospital privileges, or even career potential, should they officially seek help in managing stress, anxiety, depression, or suicidality (32). Current evidence suggests that the problem starts at early career stages. Disconcertingly, already in their first year of training, between 20 and 74% of medical residents meet the diagnostic criteria for depression (33, 34). From there, the problem seems to worsen over the years (33).

Almost all respondents to our survey had much higher perceived stress levels than average. High stress levels are not only associated with significant health concern levels (26), they are also a major factor for burnout. Burnout is defined as physical or mental collapse caused by overwork or stress. Research into the causes of burnout highlights additional risk factors such as diminishing rewards, insufficient recognition, lack of fairness, and poor relationship with communities (35). Burnout has been found to lead to health issues and poor outcomes, such as increased risk of heart disease and diabetes, professional mistakes, absenteeism, and decreased job performance (35).

The survey responses demonstrate that, on average, the mental health status of all frontline healthcare workers was fair with a mean mental health score below 40. For comparison, the average score for the United States population is 50, a full standard deviation higher (29). The comparison with a control group of Minnesota State Fair attendees who responded to the same survey tool at the same time as our healthcare workers shows the problem even more drastically. Less than 2% of State Fair attendees were in poor mental health while that was the case twenty times more often in frontline health workers.

Disturbingly, more than half of the responding physicians, and more than one-third of the responding dentists, were in poor mental health, with both genders equally affected. A number of recent studies suggest that the mental health of healthcare providers has especially suffered during the pandemic (1, 2, 16). Physicians, nurses, dentists, and other healthcare workers had to endure great risk to their own health due to the contagion. In addition, the enormous workload, financial uncertainty during times of lockdown, and growing mistrust by a portion of the population due to conspiracy theories have produced psychological pressure, depression, social anxiety, and other mental health concerns (3, 4, 7). Our cohort was affected by the same factors and it is likely that these factors took a toll on their mental health, too. Notably, all groups were equally affected and there were no significant differences in depression severity, perceived stress level, and mental health status between medical and dental frontline healthcare workers. Thus, the null hypothesis is accepted.

The healthcare industry has been disproportionally affected by the “great resignation,” and recent reports suggest that this drain is far from over. For example, nearly one in four nurses said in October 2022 they are likely to leave nursing due to COVID-19 (36). In our cohort, approximately one-third of the respondents indicated that they would rather retire or do something else for a living if they had the opportunity. It appears that a major factor is the current climate within the profession rather than the profession itself. More than half of the respondents were satisfied with their career choice; however, only few were satisfied with the current state of the healthcare industry.

What can healthcare leaders do to prevent and mitigate depression, stress, mental health issues, and burnout? It has been suggested that a multipronged approach is needed to target the problems at hand (35). The present findings corroborate this suggestion. Stehman and colleagues propose a major shift in the professional attitude and organizational policies that plague the industry (13). A 20-year-old consensus statement recommends transforming those attitudes and policies to encourage physicians to seek help for mental health issues (32). Pre-pandemic literature suggests that the path forward is a combination of individual-focused and structural strategies to meaningfully reduce burnout (37). Newer literature suggests paid time-off, mental health supports, and cognitive behavioral therapy as effective strategies (1, 38).

Our results suggest that employer-provided mental health resources, availability of counseling, and peer support groups may not be the most effective strategies. These interventions were perceived as least effective and desirable, potentially because they rely on the affected individuals seeking support. The American Medical Association has long emphasized that physicians are less likely to seek support than other professions. Many physicians are concerned about their licensure and employment opportunities since many state boards require disclosure of mental health conditions that may affect their ability to care for patients, which causes physicians to be reluctant in seeking formal care for their mental health problems. There are also the “physician personality” and training practices that program physicians to cope alone (39).

Better strategies may include adjusted work hours, rewards and incentives, and teamwork including increased clerical support. These interventions were perceived as the most effective and desirable strategies by our respondents. This could be combined with training of healthcare workers to recognize early signs of depression, stress, and burnout in their peers. This strategy was found to be promising when early-career physicians were trained to recognize early signs of distress in their colleagues (40).

This study has several limitations. First, it was limited to only two settings and therefore a numerically modest sample, which limits the generalizability of the findings. However, the chosen settings, a tertiary care medical center and a university dental school, allow new insights into the mental wellbeing of healthcare workers in both medical and dental fields. In addition, and to the best of our knowledge, the present study is the first to provide a comparison of the state of mental wellbeing between medical and dental frontline workers. Second, as survey participants were guaranteed anonymity, we relied on patient-reported outcomes and were unable to evaluate the accuracy of the reported pathologies. However, the survey contained three validated tools to gauge depression severity, level of perceived stress, and mental health status that were specifically designed for this purpose, allowing a comprehensive assessment of various aspects of mental wellbeing. Third, any survey study is limited by its response rate. A good survey response rate is generally considered to range between 5 and 30% (41). Our response rate was 32%. Finally, the study is lacking comparison data obtained before pandemic onset. For this reason, we cannot confirm that the present findings were substantially impacted by COVID-19. We refrained from asking respondents to compare their current status to their pre-pandemic status as this would have introduced a response shift. Response shift refers to measurement of patient-reported outcomes that reflect better outcomes over time not because the patient is doing better but because the patient has now adapted, psychologically, to match their new life circumstances in order to better cope with them (42). Patient-reported outcomes such as those in the present survey are particularly prone to this change over time.

## 5. Conclusion

The results suggest that the mental wellbeing of frontline health workers is poor. Medical and dental workers tend to suffer from moderate to moderately severe depression, have a much higher perceived stress level than average, and have a lower level of mental health than the general population. Many are dissatisfied with healthcare and consider leaving the industry with stress and burnout being the main reasons. Notably, all groups seem equally affected with no apparent differences between medical and dental frontline healthcare workers.

Healthcare executives and leaders must understand the gravity of the situation and take action. To improve their employees’ mental wellbeing, healthcare employers should understand their baseline, ensure co-design with staff of interventions, and track progress over time so that interventions can be changed or adjusted in case they are not working. Organizations should also pay particular attention to female workers as they constitute the largest percentage of individuals with severe depression, which may be at a higher suicide risk.

Primary, secondary, and tertiary prevention of loss of mental wellbeing in healthcare workers must become a priority. Leaders should focus on mental wellbeing before people start to struggle, not after. The results of this study may help design the tools to do so.

## Data availability statement

The raw data supporting the conclusions of this article will be made available by the authors, without undue reservation.

## Ethics statement

This study involving human participants was reviewed and approved by the Institutional Review Boards at Essentia Health Duluth and the University of Minnesota (Study number 00016700). Written informed consent for participation was not required for this study in accordance with the national legislation and the institutional requirements.

## Author contributions

TG contributed to data collection, curation, and analysis. TG and AH contributed to conceptualization, study design, data interpretation, manuscript drafting, reviewing and editing, and are responsible for the overall content. All authors contributed to the article and approved the submitted version.

## Conflict of interest

The authors declare that the research was conducted in the absence of any commercial or financial relationships that could be construed as a potential conflict of interest.

## Publisher’s note

All claims expressed in this article are solely those of the authors and do not necessarily represent those of their affiliated organizations, or those of the publisher, the editors and the reviewers. Any product that may be evaluated in this article, or claim that may be made by its manufacturer, is not guaranteed or endorsed by the publisher.
